# Conservation and diversification of the miR166 family in soybean and potential roles of newly identified miR166s

**DOI:** 10.1186/s12870-017-0983-9

**Published:** 2017-02-01

**Authors:** Xuyan Li, Xin Xie, Ji Li, Yuhai Cui, Yanming Hou, Lulu Zhai, Xiao Wang, Yanli Fu, Ranran Liu, Shaomin Bian

**Affiliations:** 1 College of Plant Science, Jilin University, Changchun, Jilin China; 2 Agriculture and Agri-Food Canada, London Research and Development Centre, London, ON Canada; 3 Department of Biology, Western University, London, ON Canada

**Keywords:** miR166 family, Soybean, Evolutionary conservation and diversification, Promoter analysis, Gene expression pattern

## Abstract

**Background:**

microRNA166 (miR166) is a highly conserved family of miRNAs implicated in a wide range of cellular and physiological processes in plants. miR166 family generally comprises multiple miR166 members in plants, which might exhibit functional redundancy and specificity. The soybean miR166 family consists of 21 members according to the miRBase database. However, the evolutionary conservation and functional diversification of miR166 family members in soybean remain poorly understood.

**Results:**

We identified five novel miR166s in soybean by data mining approach, thus enlarging the size of miR166 family from 21 to 26 members. Phylogenetic analyses of the 26 miR166s and their precursors indicated that soybean miR166 family exhibited both evolutionary conservation and diversification, and ten pairs of miR166 precursors with high sequence identity were individually grouped into a discrete clade in the phylogenetic tree. The analysis of genomic organization and evolution of *MIR166* gene family revealed that eight segmental duplications and four tandem duplications might occur during evolution of the miR166 family in soybean. The cis-elements in promoters of *MIR166* family genes and their putative targets pointed to their possible contributions to the functional conservation and diversification. The targets of soybean miR166s were predicted, and the cleavage of *ATHB14-LIKE* transcript was experimentally validated by RACE PCR. Further, the expression patterns of the five newly identified *MIR166*s and 12 target genes were examined during seed development and in response to abiotic stresses, which provided important clues for dissecting their functions and isoform specificity.

**Conclusion:**

This study enlarged the size of soybean miR166 family from 21 to 26 members, and the 26 soybean miR166s exhibited evolutionary conservation and diversification. These findings have laid a foundation for elucidating functional conservation and diversification of miR166 family members, especially during seed development or under abiotic stresses.

**Electronic supplementary material:**

The online version of this article (doi:10.1186/s12870-017-0983-9) contains supplementary material, which is available to authorized users.

## Background

MicroRNAs (miRNAs) are a class of endogenous, single-stranded, small regulatory RNA molecules, which broadly exist in diverse eukaryotes. In plants, miRNAs range from 20 to 24 nt in length and regulate the expression of target genes mainly at post-transcriptional levels [1]. Plant miRNAs are derived from their congate *MIRNA* genes that are mostly located in intergenic regions, sometimes in intragenic regions, of the plant genome [2–4]. Generally, a *MIR* gene is transcribed by Polymerase II into a capped and polyadenylated primary miRNA (pri-miRNA) [5, 6]. Subsequently, DCL protein carries out the cleavage of pri-miRNA into the stem-loop precursor (pre-miRNA), which is further processed by DCL1 to generate mature miRNA and miRNA*. Mature miRNA is then loaded into an Argonaute protein to form the miRNA-induced silencing complex (miRISC). Finally, miRISCs can bind to the transcripts of target genes through perfect or near-perfect pairing with its mature miRNA, and guild mRNA cleavage or translational inhibition in plants [7, 8]. Thus, plant miRNAs are thought to mediate almost all plant cellular and metabolic processes via regulating posttranscriptional gene silencing [9, 10].

miR166s are highly conserved in plants, and 262 miR166 species have been identified in 45 plant species according to the miRBase database (Release 21, June 2014). It is generally accepted that conserved miRNAs might play crucial roles in regulating fundamentally important biological processes [11]. So, many efforts have been made to elucidate functional roles and regulatory mechanisms of miR166 family in different plant species. To date, miR166s have been found to be involved in the modulation of various developmental processes via negatively mediating their targets, including SAM development, organ polarity, seed development, vascular patterning of shoot, root development, and nutrition ion uptake [12–17]. Additionally, some evidences indicated that miR166 family might play crucial roles in response to abiotic and biotic stresses. For example, miR166 up-regulation upon salinity stress and concomitant depression of its targets were observed in andigena potato, suggesting an important network node for modulating gene expression responsive for growth adjustments [18]. Similarly, miR166 was induced by *Phytophthora sojae* infection in soybean, indicating that it may be implicated in soybean basal defense [19].

Plants often harbor a number of miR166s derived from multigene family with the individual loci. For example, there are 21 miR166s in soybean, 17 in *Populus trichocarpa*, 9 in *Arabidopsis thaliana*, 13 in maize, rice, and *Physcomitrella patens* according to miRBase database (Release 21, June 2014). The miR166s in multigene family were found to be highly conserved to target *HD-ZIP III* family genes such as *REVOLUTA* (*REV*), *PHABULOSA* (*PHB*), *PHAVOLUTA* (*PHV*), *CORONA* (*CNA*) and *ATHB8* in a broad range of plant species, indicating that they might exhibit high degree of functional redundancy [20–23]. Nevertheless, emerging evidences indicate that functional specialization exists in miR166 family. Firstly, miR166s in different species can be predicted and/or demonstrated to target *non-HD-ZIPIII* genes. For example, *RICE Dof DAILY FLUCTUATIONS 1* (*RDD1*) can be targeted by miR166 in rice, thereby regulating nutrient ion uptake and accumulation in rice [16]; while miR166m rather than other miR166 members in *Physcomitrella patens* was predicted to target 3 *non-HD-ZIPIII* transcripts such as TC21828, FC366912 and TC13986 [24]. These different set of targets suggest functional diversity of miR166 family in plant species. Secondly, spatio-temporal expression patterns of *MIR166* genes also reflect their functional specialization [25]. A good example is that only five members of *MIR166* gene family (*MIR165a*, *MIR165b*, *MIR166a*, *MIR166b* and *MIR166g*) in *Arabidopsis* were found to be expressed in embryo, which might provide a positional signal from the basal–peripheral region of early embryos, whereas the other four *MIR166*s can not be detected in any stage of embryogenesis [13]. Similarly, three *MIR166* genes (*MIR165a*, *MIR166a* and *MIR166b*) were observed to be expressed specifically in the root endodermis and post-embryonic meristem in *Arabidopsis* [26]. Additionally, phylogenetic analysis revealed diversification of *MIR166* gene family among *Arabidopsis*, rice and *Physcomitrella patens*, which laid a foundation for further exploration of the evolutionary and functional divergence of plant miRNAs [24]. Evidently, the existence of multiple copies of *MIR166* genes contributes to the functional diversity and specificity of *MIR166* gene family members in plants. Thus, addressing the evolutionary conservation and diversification of *MIR166* gene family becomes an important step towards understanding their fine-tuned regulatory roles in plant developmental processes and/or resistance to stresses.

Soybean harbors the largest miR166 family with 21 members in plant. Although the co-evolution and characteristics of *MIRNA* genes were globally investigated in soybean [27–29], the evolutionary conservation and functional diversification of *MIR166* family members in soybean remain poorly understood. In this study, five novel miR166s in soybean were identified by data mining approach. Subsequently, the genomic organization and evolution of all the 26 *MIR166* genes were investigated; and as a result, eight segmental duplications and four tandem duplications were found to have possibly occurred during evolution of *MIR166* gene family in soybean. Furthermore, promoter analysis and target prediction results pointed to functional diversification of soybean *MIR166* gene family. Finally, the expression patterns of the five newly identified *MIR166*s were examined during seed development and in response to abiotic stresses. These findings laid a foundation for elucidating functional diversification of soybean *MIR166* gene family, especially during seed development or in response to abiotic stresses.

## Results

### Identification of novel miR166s in soybean

To identify novel miR166s in soybean, pre-miR165/166 sequences from soybean, *Arabidopsis* and rice were used as queries to conduct BLAST search against the soybean genomic database (http://www.phytozome.net/). Subsequently, the matched genomic sequences were analyzed following a series of screening criteria for encoding miRNA sequence, and five novel pre-miR166s were identified in soybean. Based on soybean miR166 nomenclature (gma-miR166a-u) in miRBase (Release 21, 2014), the five newly identified miR166s were orderly named as miR166v-z.

**Fig. 1:**
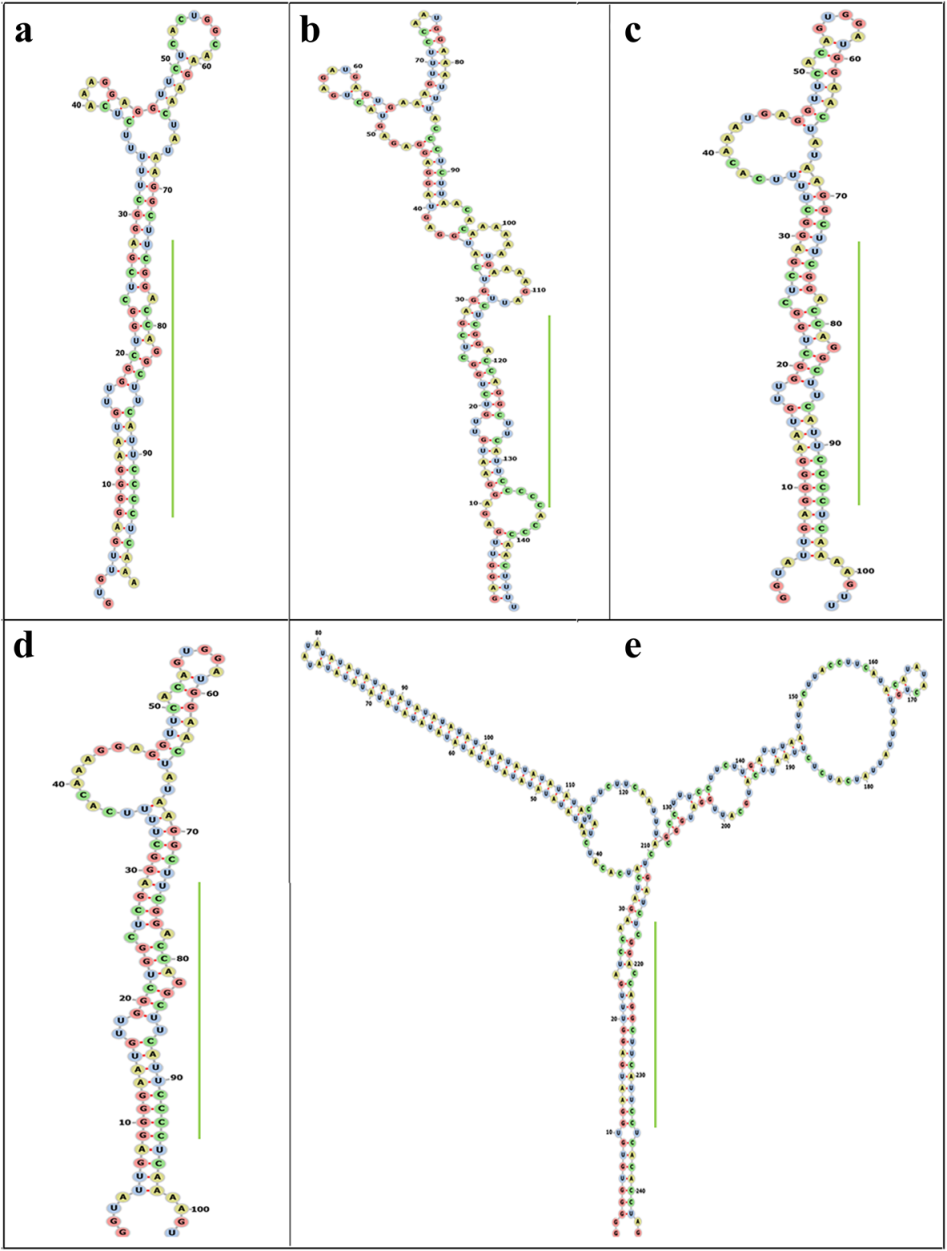
Stem-loop structures of the five newly identified pre-miR166s in soybean. The panel **a**-**e** sequentially correspond to pre-miR166v-z. The mature miRNA portion is highlighted in *green bar*

The characteristics of the five newly identified miR166s were summarized in Tables 1 and 2. All the five newly identified mature miR166 sequences were identical to their paralogs in soybean. Also, they were located on the 3’arm of the secondary stem-loop hairpin structure of the pre-miRNAs (Fig. 1). The nucleotide lengths of the five newly identified pre-miRNAs ranged from 98 to 244 nt, which fall right within the normal range of plant miRNA precursors (55 to 930 nt, mean = ~146 nt) [30]. Negative minimal folding free energy (MFE) is an important criterion for distinguishing the miRNA from other types of RNA [31]. In the study, all the potential miRNA precursors exhibited low MFEs ranging from -46.0 to -90.7 kcal/mol (Table 2). Furthermore, to normalize the potential effect of sequence length on MFEs, the minimal folding free energy index (MFEI) was calculated to differentiate miRNAs from other RNAs. As shown in Table 2, the pre-miRNAs had a high MFEI (0.71–1.35) with an average of about 0.96, suggesting that they are very likely to be miRNA precursors. Thus, the size of soybean miR166 family was enlarged from 21 to 26 members (Additional file 1: Table S1).

**Table 1:** Characteristics of the five newly identified miR166s in soybean

ID	miR Sequence	LM^a^	Arm	Homologue	NM^b^
miR166v	UCGGACCAGGCUUCAUUCCCC	21	−3’	gma-miR166a-g,i,n,o	0
miR166w	UCGGACCAGGCUUCAUUCCCC	21	−3’	gma-miR166a-g,i,n,o	0
miR166x	UCGGACCAGGCUUCAUUCCCC	21	−3’	gma-miR166a-g,i,n,o	0
miR166y	UCGGACCAGGCUUCAUUCCCC	21	−3’	gma-miR166a-g,i,n,o	0
miR166z	UCUCGGACCAGGCUUCAUUCC	21	3’	gma-miR166k,h	0

^a^Length of miRNA; Arm = location of mature miRNAs on secondary stem-loop structures of pre- miRNA sequences; ^b^Number of mismatch between predicted and homologous miRNA

**Table 2:** Characteristics of the five newly identified pre-miR166s in soybean

ID	LP^a^	Chrom-osome	Location	U content	MFE kcal/mol	MFEI	NM^b^	G_U pairs
pre- miR166v	98	Chr06	12993196–12993098(-)	0.273	−46.60	0.93	3	2
pre- miR166w	147	Chr01	26215847–26215701(-)	0.252	−46.30	0.71	4	2
pre- miR166x	103	Chr08	282750–282648(-)	0.272	−46.20	0.92	3	2
pre- miR166y	102	Chr05	37747561–37747460(-)	0.414	−90.70	1.35	2	3
pre- miR166z	244	Chr16	3661371–3661614(+)	0.255	−46.00	0.90	3	2

^a^length of pre-miRNA; ^b^number of mismatches between predicted miRNA and miRNA*

### Evolutionary relationship of Pre-miR166s in soybean

**Fig. 2:**
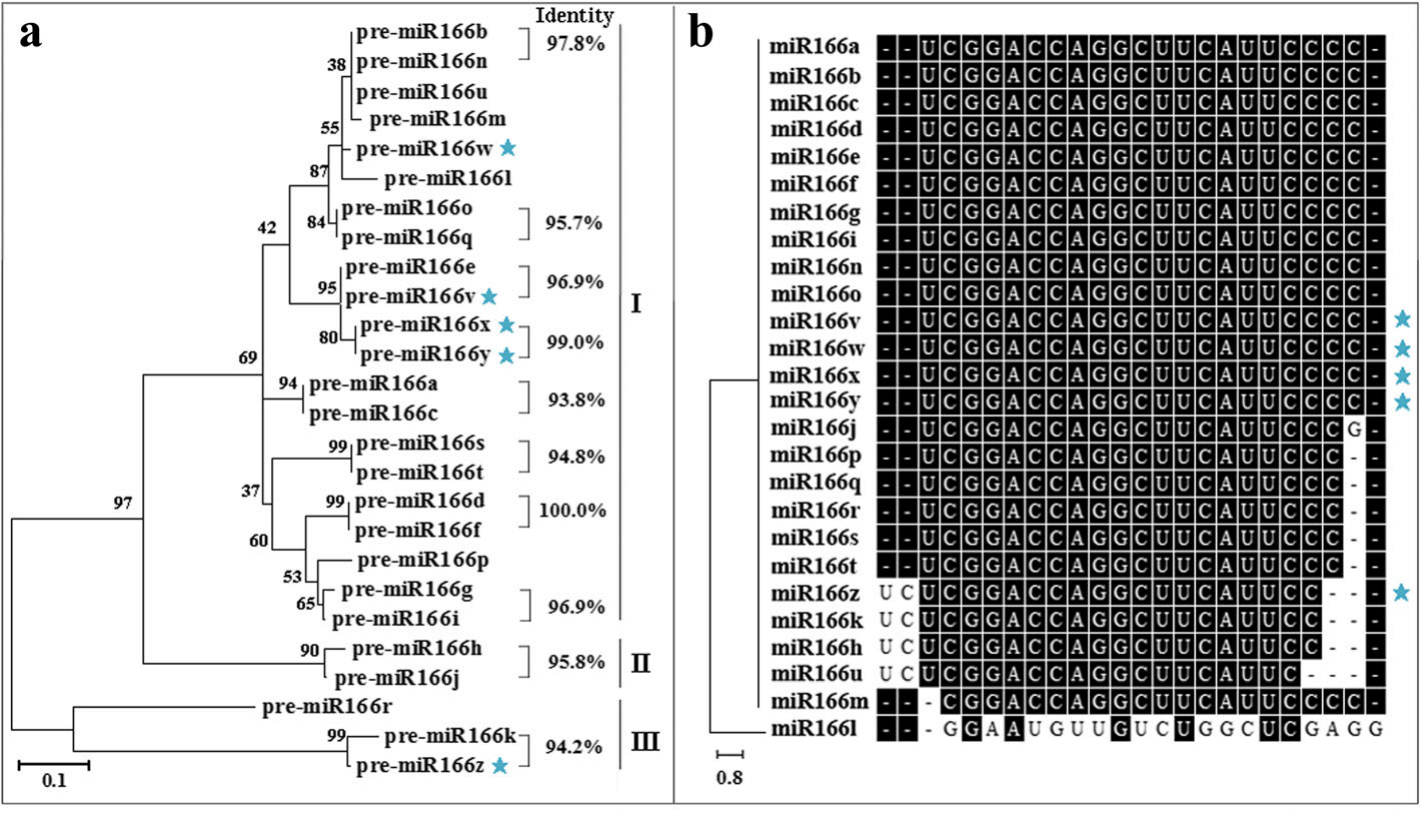
Phylogenetic analysis and alignment of miR166 family in soybean. **a** Phylogenetic analysis of miR166 precursors. **b** Phylogenetic analysis and alignment of mature miR166s. The five newly identified miR166s in soybean are highlighted with a *star*, and the identity between paralogous pair is listed

To examine the evolutionary relationship among the 26 miR166s in soybean, phylogenetic analysis was conducted using the sequences of their precursors and mature miRNAs, respectively. The phylogram of miR166 precursors revealed that 26 soybean pre-miR166s were grouped into three classes representing similarities and divergence. As shown in Fig. 2a, 21 pre-miR166s formed the largest class, and the rest of pre-miR166s were clustered into two separate branches away from the majority of pre-miR166s. The five newly identified pre-miR166s (pre-miR166v-z) were unevenly distributed in the phylogenetic tree. As indicated in Fig. 2a, pre-miR166w was grouped together with pre-miR166b, n ,u, m, while pre-miR166v, pre-miR166x and pre-miR166y formed a branch with miR166e in class I. In contrast, pre-miR166z formed a separate branch with pre-miR166k in class III, suggesting that the newly identified pre-miR166s might have evolutionary diversification. It is interesting to note that ten pairs of miR166 precursors with high sequence identity (93.8% or higher), were individually grouped into discrete clades in the phylogenetic tree (Fig. 2a and Additional file 2: Figure S1), including three pairs containing the newly identified pre-miR166s (pre-miR166v and pre-miR166e, pre-miR166x and pre-miR166y, pre-miR166z and pre-miR166k) and seven previously known pre-miR166 pairs (pre-miR166b and pre-miR166n, pre-miR166a and pre-miR166c, pre-miR166s and pre-miR166t, pre-miR166d and pre-miR166f, pre-miR166g and pre-miR166i, pre-miR166h and pre-miR166j, pre-miR166o and pre-miR166q). These observations suggest that these pairs of pre-miR166s are evolutionarily closer to each other compared with the rest members in miR166 family.

Meanwhile, the topological tree of mature miR166s strongly supported the conserved property of miR166 family. As shown in Fig. 2b, 26 mature miR166s were grouped into two clades, and all the mature miR166s except miR166l showed identical or nearly identical sequence. To further understand the conservation and divergence of soybean miR166s, 63 unique mature miR166 sequences were isolated from miRBase (Release 21) and designated as UmiR166-1 to 63, respectively (Additional file 3: Table S2). The phylogenetic analysis indicated that the 63 UmiR166s were clustered into four classes representing conservation and divergence (Additional file 4: Figure S2). Six UmiR166s presentative of 26 soybean miR166s were grouped in the Class IV (such as miR166a-g, i, n, o, v, w, x, y, UmiR166-28; miR166p-t, UmiR166-29; miR166m, UmiR166-41; miR166z, k, h, UmiR166-49; miR166j, UmiR166-53; miR166u, UmiR166-62), while the representative of gma-miR166l (UmiR166-13) formed a branch with 26 UmiR166s in the Class I. The observation indicated that soybean miR166s are highly conserved among plant species. We further performed Kalmogorov-Smirnov analysis to assess the percentage identity of soybean miR166s and their precursors, respectively. As shown in Figure S3 (Additional file 5), ~0.25 fraction of mature miR166s had more than 80% sequence identity, whereas ~0.25 fraction of pre-miR166s only showed ~20% sequence identity, indicating that mature miR166s are more conserved than their precursors. It was also observed that each of the pre-miR166 paralogous pairs generated identical mature sequence except the pair of miR166h and miR166j, suggesting that they might target the same set of genes in soybean.

### Chromosomal distributions and duplications of *MIR166*s in soybean

**Fig. 3:**
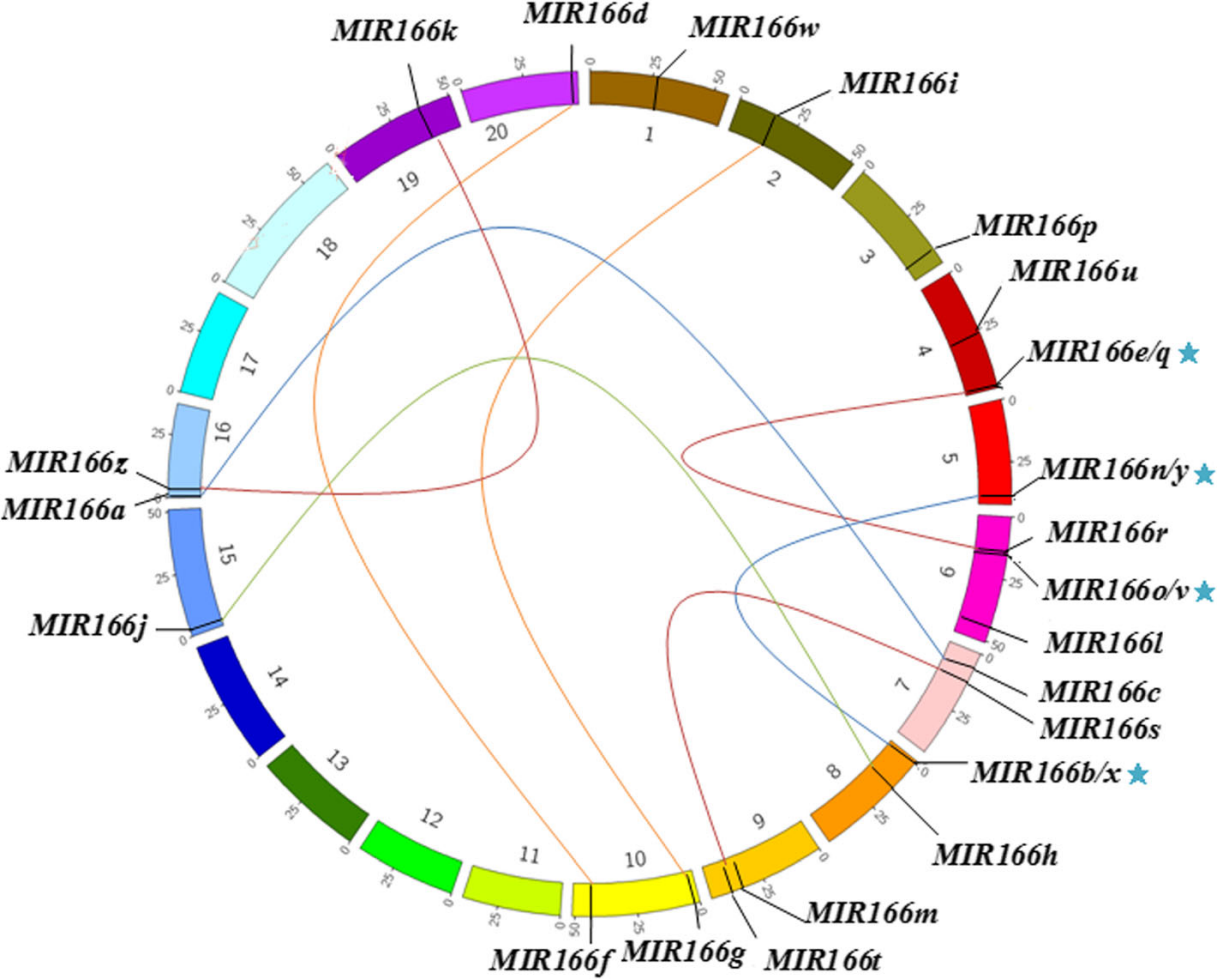
Chromosomal localization and duplication of *MIR166* family genes in soybean. Each *colored box* represents a chromosome. The approximate distribution of each soybean *MIR166* gene is marked on the *circle* with a short *black line*. Tandem duplication clusters are indicated with *star. Colored lines* indicate the linkage group with segmental duplicated *MIR166* genes, and segmental duplication regions were determined using the Plant Genome Duplication Database

To determine the distribution of *MIR166*s on different chromosomes in soybean, a chromosome map was constructed. As shown in Fig. 3, the 26 pre-miR166s are unevenly located on 14 different chromosomes in soybean. Chromosome 6 has the largest number with four pre-miR166s, followed by chromosome 4 and 8 with three pre-miR166s, while two miR166s were distributed on chromosome 5, 7, 9, 10, 16, and one miR166 on chromosome 1, 2, 3, 15, 19 and 20. Further examination revealed that 12 pre-miR166s were found in the intergenic regions of the genome. Intriguingly, 14 intragenic miR166s were mapped to the region of intron, exon or/and UTR in host genes (Additional file 6: Table S3). For example, pre-miR166p and pre-miR166l were situated in the UTR region of the *UNAG KINASE* gene and anti-sense strand of intron region of the *CHAPERONIN* gene, respectively. These results implied that interactions might exist between intragenic miR166s and host genes.

It has been demonstrated previously that approximately 20% of plant miRNAs are clustered, and generally contain conserved miRNAs of the same family [32]. To estimate cluster numbers in soybean miR166 family, a maximal distance of 3 kb between two consecutive miR166s was used as a stringent criterion. As a result, four miR166 clusters on the same DNA strand were identified in soybean (Fig. 3), and each cluster contained two copies of miR166 family members (pre-mir166v and pre-miR166o, pre-miR166x and pre-miR166b, pre-miR166y and pre-miR166n, pre-miR166e and pre-mir166q). The distance between two pre-miR166s within each cluster ranged from 90 to 158 nt (Additional file 1: Table S1), suggesting that they might be polycistronic precursors. To confirm their polycistronic nature, data mining was conducted against soybean Expressed Sequence Tags (EST) database since ESTs could provide evidence for *MIR* gene expression in different tissues or processes. As shown in Table S3 (Additional file 6), the corresponding EST simultaneously contained the sequences of two pre-miR166s that belong to the same cluster. These observations indicated that the corresponding *MIR* genes can indeed be transcribed into polycistronic precursors carrying two tandem copies of miR166s. Thus, we tentatively named these genes as *MIR166e/q*, *MIR166v/o*, *MIR166x/b* and *MIR166y/n*, and three of the five newly identified pre-miR166s belonged to polycistronic precursors. It has been reported that homologous *MIR* gene clusters are associated with dosage effect [33]. Thus, existence of homologous miR166s from a polycistronic *MIR* gene implied that they might exert dosage effect on the regulation of target gene expression.

Based on coordinates of pre-miR166s or their neighbor genes (Additional file 1: Table S1), we further investigated whether traceable genome duplications contributed to the expansion of *MIR16*6 gene family in soybean. Eight sets of *MIR166s* were mapped on eight distinct duplicate blocks (Additional file 7: Table S4), including three newly identified miR166-containing blocks (*MIR166v/o* and *MIR166e/q* on the block 446, *MIR166x/b* and *MIR166y/n* on the block 571, *MIR166z* and *MIR166k* on the block 1447) and five previously known miR166-containing blocks (*MIR166g* and *MIR166i* on the block 210, *MIR166s* and *MIR166t* on the block 816, *MIR166a* and *MIR166c* on the block 867, *MIR166h* and *MIR166j* on the block 958, *MIR166d* and *MIR166f* on the block 1157). The analysis suggested that these pairs of *MIR166*s on the same block were possibly derived from segmental duplication events during evolution. To investigate the selective evolutionary pressure on *MIR166* gene divergence after duplication, the non-synonymous/synonymous substitution ratio (Ka/Ks) was retrieved for the eight duplicated pairs of *MIR166* genes. As shown in Additional file 7: Table S4 the Ka/Ks value of all the duplicated gene pairs are less than 1, suggesting that these genes might have undergone a purifying selection with limited functional divergence after duplication [34].

### Analyzing cis-regulatory motifs in *MIR166* promoters

**Fig. 4:**
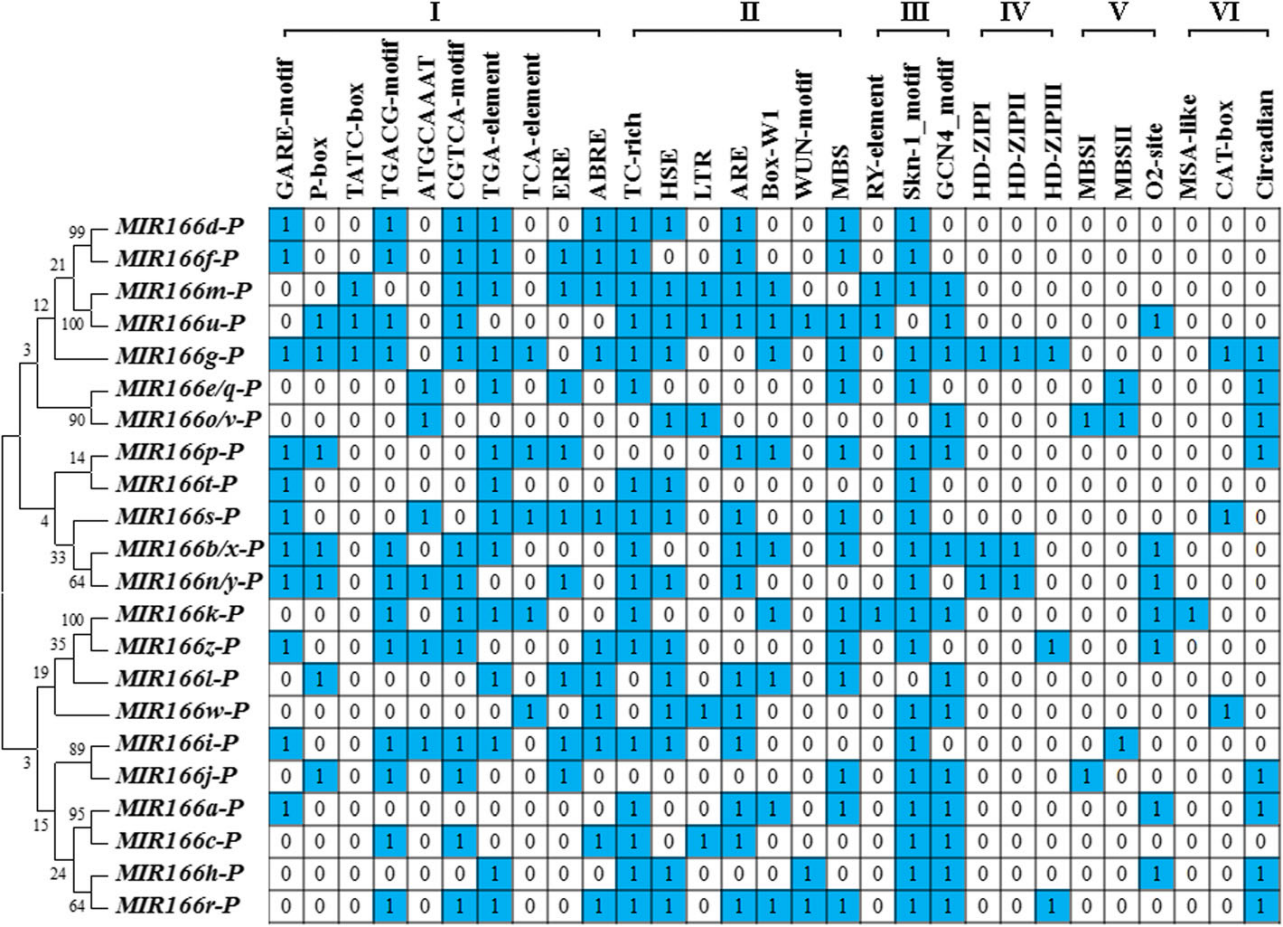
Promoter analysis of *MIR166* genes in soybean. Phylogenetic relationship of *MIR166* promoters is shown in left panel. The 6 classes of cis-elements (I-VI) are responsive to hormones, abiotic stresses, seed development, HD-ZIPIII binding, biosynthesis and metabolism, cell cycle and circadian, respectively. If *MIR166* harbors the cis-element in promoter region, the *box* is labeled with the number 1 and highlighted in *blue* color, otherwise the *box* is labeled with the number 0

To provide clues for understanding the regulatory mechanism of *MIR166* expression, bioinformatics analysis was conducted to identify putative cis-regulatory elements in promoter region, which was defined based on the estimated transcription start site (TSS). As shown in Additional file 8: Table S5 most putative promoters were located within 800 bp upstream region of pre-miRNA foldbacks, consistent with previous report that the distance between TSS and precursor is less than 1 kb for more than 85% miRNAs in *Arabidopsis* [35]. As expected, the core promoter element TATA-box was observed in all the *MIR166* promoter regions, while 18 out of 22 *MIR166* genes contained CAAT-box, a common cis-acting element in promoter and enhancer regions. Furthermore, specific promoter elements were analyzed for understanding *MIR166* functional roles. As indicated in Additional file 9: Table S6 the specific promoter elements were classified into eight classes, including light response, hormone response, seed development, leaf development, biosynthesis and metabolism, cell cycle and circadian, biotic or abiotic defense, and others. The cis-regulatory elements were non-uniformly distributed in different *MIR166* promoters. Light-responsive elements were most enriched in the promoters of *MIR166* family genes (100% of promoters were found to contain light responsive elements), followed by two endosperm-specific regulatory motifs (Skn1, 84.6% and GCN4, 65.4%) and four stress-responsive elements such as TC-rich (76.9%), HSE (61.5%), ARE (61.5%) and MBS (61.5%), suggesting that these cis-elements are fundamental to the expression of *MIR166* family genes. In contrast, some motifs exist only in three or fewer members of *MIR166* gene family (such as MSA-like 3.8%, RY-element 11.5%, HD-ZIP III binding site 11.5% and MBSI 11.5%), implying that these motifs might be involved in controlling the specificity of *MIR166* expression. It has been well demonstrated that miR166 family can control the expression of *HD-ZIPIII* genes via directly targeting their transcripts for degradation [14, 17, 21]. Interestingly, *MIR166g*, *MIR166r* and *MIR166z* contained HD-ZIPIII binding site in their promoter regions (Fig. 4), implying a possible HD-ZIPIII-mediated feedback regulatory loop for *MIR166* expression in soybean.

To investigate the evolutionary relationship between the promoters of *MIR166* family genes, phylogenetic analysis was also conducted. As shown in Fig. 4, nine pairs of *MIR166* promoters can individually form a discrete clade in phylogenetic tree (*MIR166d* and *MIR166f*, *MIR166m* and *MIR166u*, *MIR166e/q* and *MIR166o/v*, *MIR166p* and *MIR166t*, *MIR166b/x* and *MIR166n/y*, *MIR166k* and *MIR166z*, *MIR166i* and *MIR166j*, *MIR166a* and *MIR166c*, *MIR166h* and *MIR166r*) and have similar motif composition in their promoter regions.

### Expression analysis of *MIR166*s in soybean

**Fig. 5:**
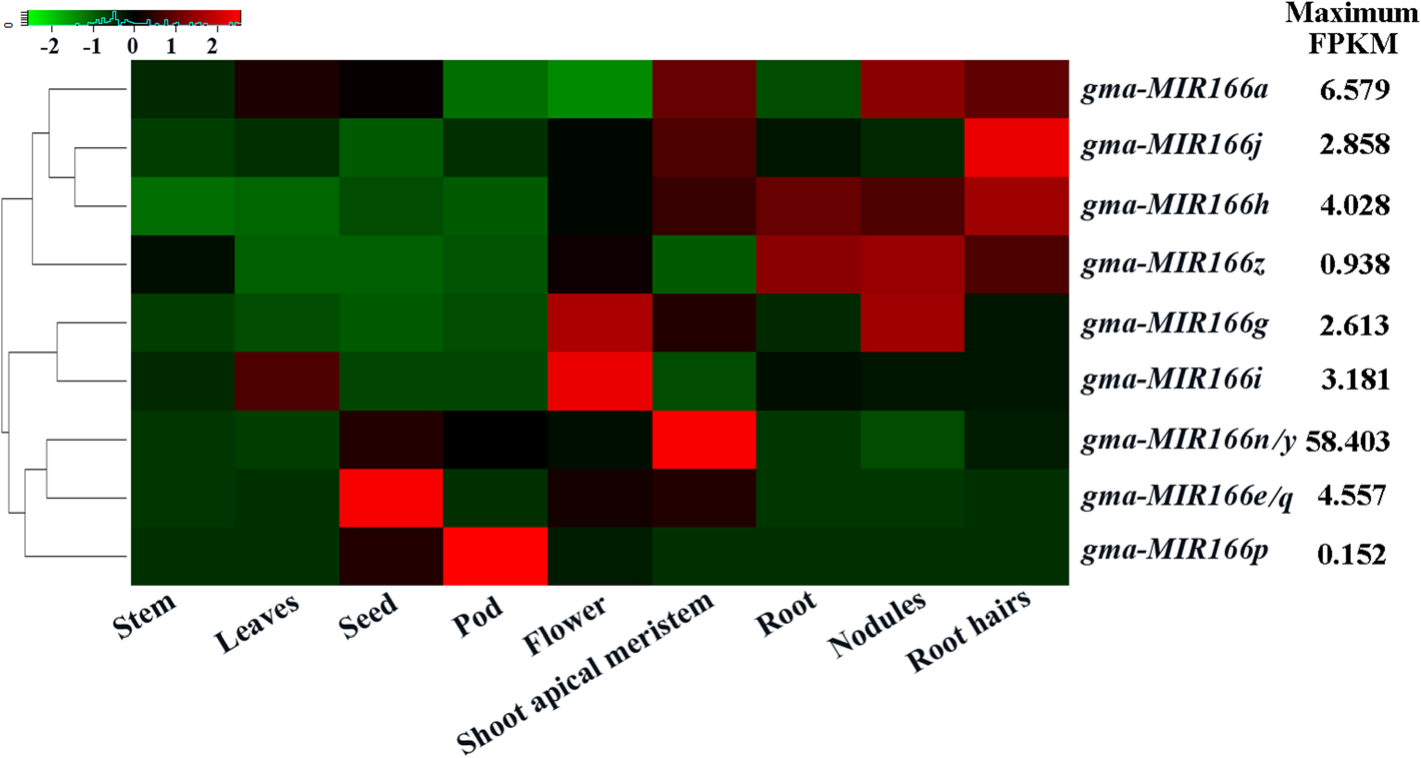
Expression analysis of soybean *MIR166* genes in various tissues. The transcript profiling data of soybean tissues were extracted from the publicly-available Phytozome database (http://www.phytozome.net) for heatmap generation. The color scale above the heat map indicates gene expression levels, low transcript abundance indicated by *green* color and high transcript abundance indicated by *red* color. Maximum FPKM value for each *MIR166* is shown

To investigate the expression patterns of *MIR166* genes, we mined the publicly available transcript profiling data of soybean tissues at the Phytozome database (http://www.phytozome.net) and found that 9 *MIR166* genes showed tissue-specific expression patterns. Based on their expression patterns, the 9 *MIR166* genes can be categorized into three groups (Fig. 5). Group 1 comprises *MIR166a*, *MIR166j*, *MIR166h* and *MIR166z*, which were mainly expressed in root, root hair, nodule and/or shoot apical meristem. Group 2 contains *MIR166g* and *MIR166i* with high expression in flowers, shoot apical meristem, leaf and/or nodules, while group 3 consists of *MIR166n/y*, *MIR166e/q* and *MIR166p* with high transcript abundance either in seed, pod, flower or shoot apical meristem. The maximum fragments per kilobase of transcript per million mapped reads (FPKM) for *MIR166n/y* was higher (58.403) compared to the reads for the rest of *MIR166* gene family (0.152 to 6.579), suggesting that *MIR166n/y* might perform a major function among *MIR166* family genes in soybean.

Additionally, blast search against ESTs in NCBI database revealed that 12 pre-miR166s can perfectly match with one or more ESTs including nine gene-mapped pre-miR166s and three gene-unmapped pre-miR166s (Additional file 6: Table S3). Among them, three ESTs generated from *MIR166e/q*, *MIR166b/x*, *MIR166o/v* were derived from immature seeds, and one EST for miR166j from germinating shoot. Additionally, five ESTs generating pre-miR166a, i, n, r, y were originated from the tissues under abiotic stresses. These observations not only confirmed that at least 12 pre-miR166s (including four of the newly identified pre-miR166s), out of the 26 soybean miR166s, are indeed transcribed, but also implied that these miR166s might probably be involved in seed development, germination and/or in response to abiotic stresses.

### Targets of miR166s in soybean

**Fig. 6:**
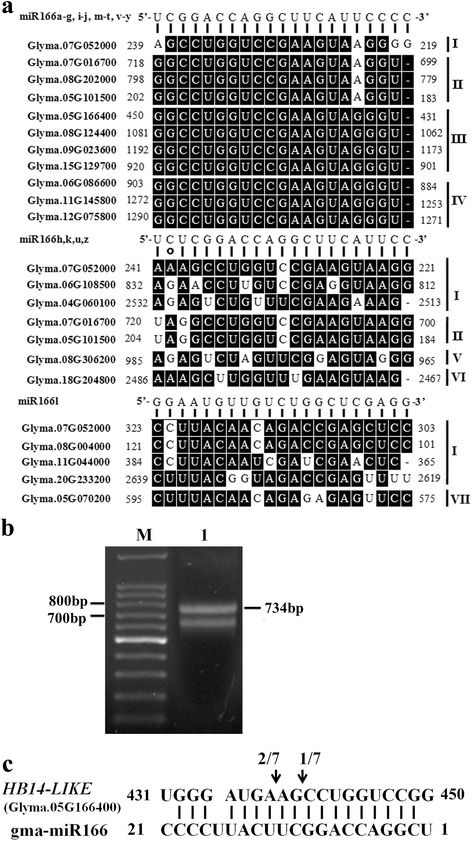
Predicted targets of miR166 family and validation of miR166 target gene using RLM-RACE PCR. **a** The alignments of fragments of target mRNAs that have complementarity to miR166s are shown. miR166 sequences are displayed to highlight complementarity to target mRNAs. The targets are classified into seven groups (I, no annotation; II, *ATHB-15*; III, *ATHB14-LIKE*; IV, *REVOLUTA-LIKE*; V, *PHYTOENE SYNTHASE*; VI, *AMMONIUM TRANSPORTER 2-LIKE*; VII, two-component response regulator-like *APRR2*). **b** RLM-RACE PCR for *ATHB14-LIKE*. Lane 1 and M show cleaved product of desired size (734 bp) and DNA ladder, respectively. **c** Mapping of *ATHB14-LIKE* mRNA cleavage sites by RLM-RACE. The *arrows* indicate the cleavage sites and the numbers show the frequency of clones sequenced

To obtain a further understanding of biological functions of miR166 family in soybean, the transcripts of *Glycine max* from JGI genomic project were utilized as a reference system to predict the targets of soybean miR166s by employing the psRNATarget tool. Using three representative mature miR166s as query sequences, 19 target genes were identified. As shown in Fig. 6a, 21 miR166s (miR166a-g,i-j,m-t,v-y) were predicted to target ten members of the *HD-ZIPIII* family genes (4 *ATHB-14-LIKE*, 3 *HB-15*, 3 *REVOLUTA-LIKE*), whereas miR166h,k,u,z,l might target the transcripts other than *HD-ZIP III* family genes, such as *PHYTOENE SYNTHASE* (*PSY*), *AMMONIUM TRANSPORTER 2-LIKE* (*AMT2-LIKE*) and/or two-component response regulator-like *APRR2*. This results support the previous report that miR166 family displays the functional diversity and specificity in plants [24]. Among the predicted targets, cleavage of *ATHB14-LIKE* transcript (Glyma05G166400) was chosen to be validated using RLM-RACE approach. As shown in Fig. 6b-c, the product of *ATHB14-LIKE* was precisely cleaved at the 10th or 12th position of complementarity from the 5’ end of miR166s, indicating that miR166s can be indeed involved in the regulation of biological processes via targeting *HD-ZIP III* family in soybean.

**Fig. 7:**
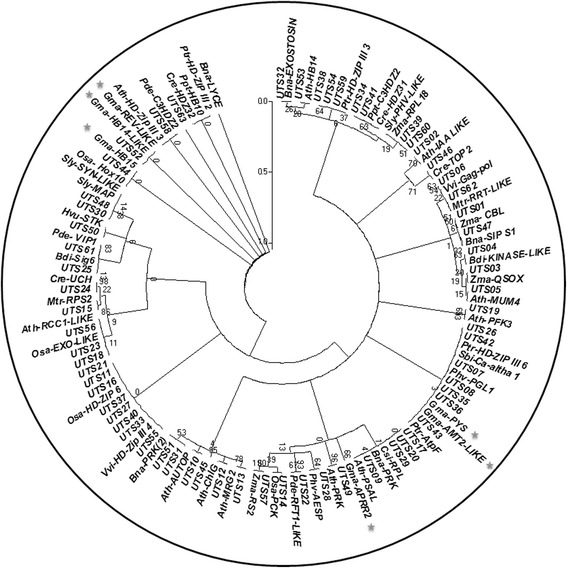
Phylogenetic analysis of unique target sequences (UTS) present in plant species and binding sites of miR166 in target genes. Target genes of miR166s in soybean are highlighted with a *star*. The details of UTSs were listed in Additional file 10: Table S7

To understand the evolutionary relationship between miR166s and their target genes, phylogenetic analysis were performed using 63 unique target sequences (UTS) and 52 target genes from diverse plant species (Additional file 10: Table S7). As shown in Fig. 7, 28 out of 63 UTSs were clustered with *HD-ZIP* family in the phylogenetic tree. It is reasonable since *HD-ZIP III* family is highly conserved targets of miR166s among diverse plant species. Further observation pointed that UTS52 and UTS44 were very close to *HD-ZIP III* family genes in soybean including *gma-HB14-LIKE*, *gma-REV-LIKE* and *gma-HB15*, indicating highly conserved binding site of mi166s in soybean *HD-ZIP III* family. In contrast, two non-*HD-ZIP III* target genes in soybean (*gma-PSY* and *gma-ATM2*) had seven closely related UTSs, which were also close to *sbi-Calcium channel alpha-1 subunit* (*sbi-Ca-alpha 1*), *ptr-HD-ZIP III 6*, *phv-Plastoglobulin-1* (*phv-PLG*), *Ptr-ATP synthase B chain AtpF*. Similarly, *gma-APPR2* was grouped with 4 UTSs (UTS09,17,20,29) and some target genes including *Csi-Resistance protein-like protein* (*csi-RPL*), *Bna-PRK*, *Ath-Photosystem I reaction center subunit XI* (*Ath-PSAL*). The observations suggested that *non-HD-ZIP III* target genes are diverse among different plant species.

**Fig. 8:**
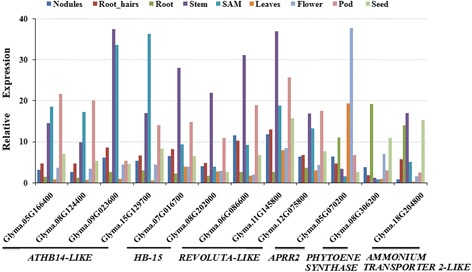
Expression patterns of miR166 target genes in different soybean tissues. The transcript profiling data of 12 annotated target genes in soybean tissues were extracted from the publicly-available Phytozome database (http://www.phytozome.net)

To provide clues for their functions during soybean development, the expression patterns of 12 annotated target genes of miR166s were extracted from genome-wide transcript profiling data of soybean tissues from the Phytozome database. As shown in Fig. 8, the members of the *HD-ZIPIII* family were highly expressed in stem, shoot apical meristem and/or pods, whereas low transcript accumulation was observed in roots and leaves. Additionally, the transcript accumulation of *APRR2*, *PSY* and *AMT2-LIKE* was highest in flower, root and stem, respectively.

### Expression pattern of the five newly identified *MIR166* genes during seed development or in response to abiotic stresses

Several studies have documented that miR166 plays important roles during seed development and in response to abiotic stresses [13, 14, 36–38]. To provide clues for their functional roles in soybean, we took the five newly identified pre-miR166s as examples to examine their expression patterns during seed development and in response to cold, drought and salinity stresses.

**Fig. 9:**
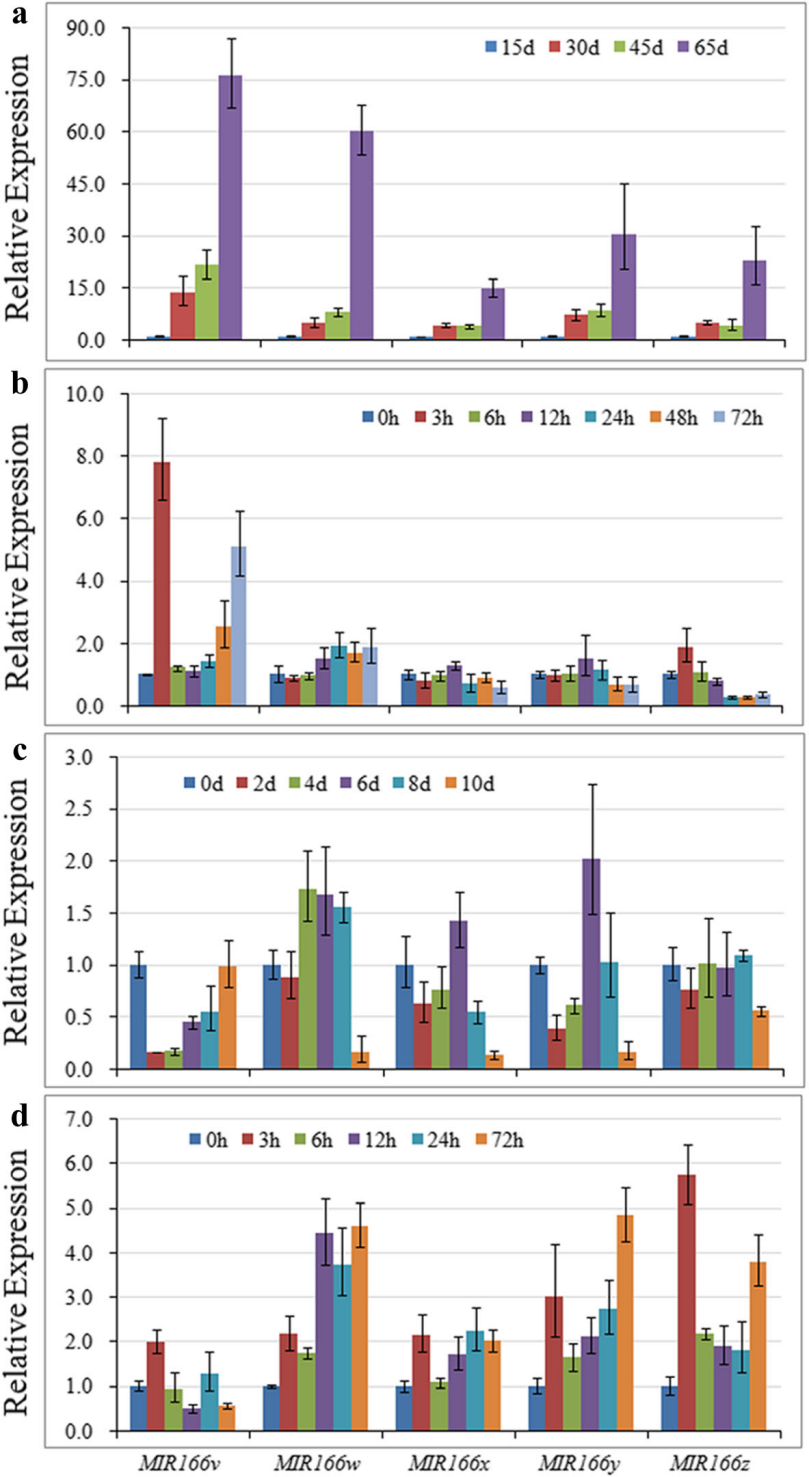
Expression analysis of the five newly identified *MIR166* genes in response to seed development and abiotic stresses. **a** Expression patterns of the 5 *MIR166* genes during seed development (15, 30, 45, 65 days after flowering). **b** Expression patterns of the 5 *MIR166* genes in seedlings exposed to cold stress for 0, 3, 6, 12, 24, 48 and 72 h. **c** Expression patterns of the 5 *MIR166* genes in seedlings exposed to drought stress for 0, 2, 4, 6, 8 and 10 d. **d** Expression patterns of the 5 *MIR166* genes in seedlings exposed to salinity stress for 0, 3, 6, 12, 24 and 72 h. Error bars indicate SE of two biological and three technical replicates. Values were normalized against the *SUBI3* gene

Expression patterns of the five newly identified *MIR166*s were investigated using seeds collected at 15, 30, 45, 65 days after flowering (DAF). As shown in Fig. 9a, all the five newly identified *MIR166* genes showed gradual increase in transcript accumulation as seeds developed, and reached the peak at 65 DAF. Their fold changes were up to 76.31, 60.34, 14.84, 30.28 and 22.91, respectively, compared with the ones at 15 DAF, suggesting that the five newly identified *MIR166*s might play an important role at late stages of seed development.

Ten-day-old soybean seedlings were exposed to cold stress at 4 °C for 0, 3, 6, 12, 24, 48 and 72 h, and expression of the five newly identified *MIR166*s were monitored. As indicated in Fig. 9b, the expression of *MIR166v* and *MIR166z* were dramatically increased by 7.79- and 1.88-fold, respectively, at 3 h after cold treatment, implying that *MIR166v* and *MIR166z* might respond to cold stress at early stage. *MIR166w* showed gradual increase in transcript accumulation as the stress prolonged, while *MIR166x* and *MIR166y* exhibited slight decrease at late stage of cold stress.

When soybean seedlings were exposed to drought stress, the expression of *MIR166v* and *MIR166y* was promptly decreased by 6.14 and 2.60 folds, respectively, at 2 days after treatment (Fig. 9c), indicating that they might be relatively sensitive to drought stress. Also, a distinct decrease in transcript accumulation after 10 days of drought treatment was observed for *MIR166w*, *MIR166x*, *MIR166y* and *MIR166z* with 6.37, 7.75, 6.06 and 1.80 fold changes, respectively as compared to control.

When young seedlings were subjected to salt stress, the expressions of all the 5 *MIR166* genes were promptly increased at 3 h after salt treatment (Fig. 9d). Especially, the expression of *MIR166v* and *MIR166z* reached their maximum level at 3 h salt stress with 1.99 and 5.73 fold changes, respectively as compared to non-salinity control, suggesting that they might be responsive to salt stress at early stage. *MIR166w* and *MIR166y* showed gradual increase in transcript accumulation as the stress prolonged, and reached their maximum level at 72 h salt stress with 4.59 and 4.82 fold changes, respectively as compared to control.

### Expression pattern of miR166 target genes during seed development or in response to abiotic stresses

**Fig. 10:**
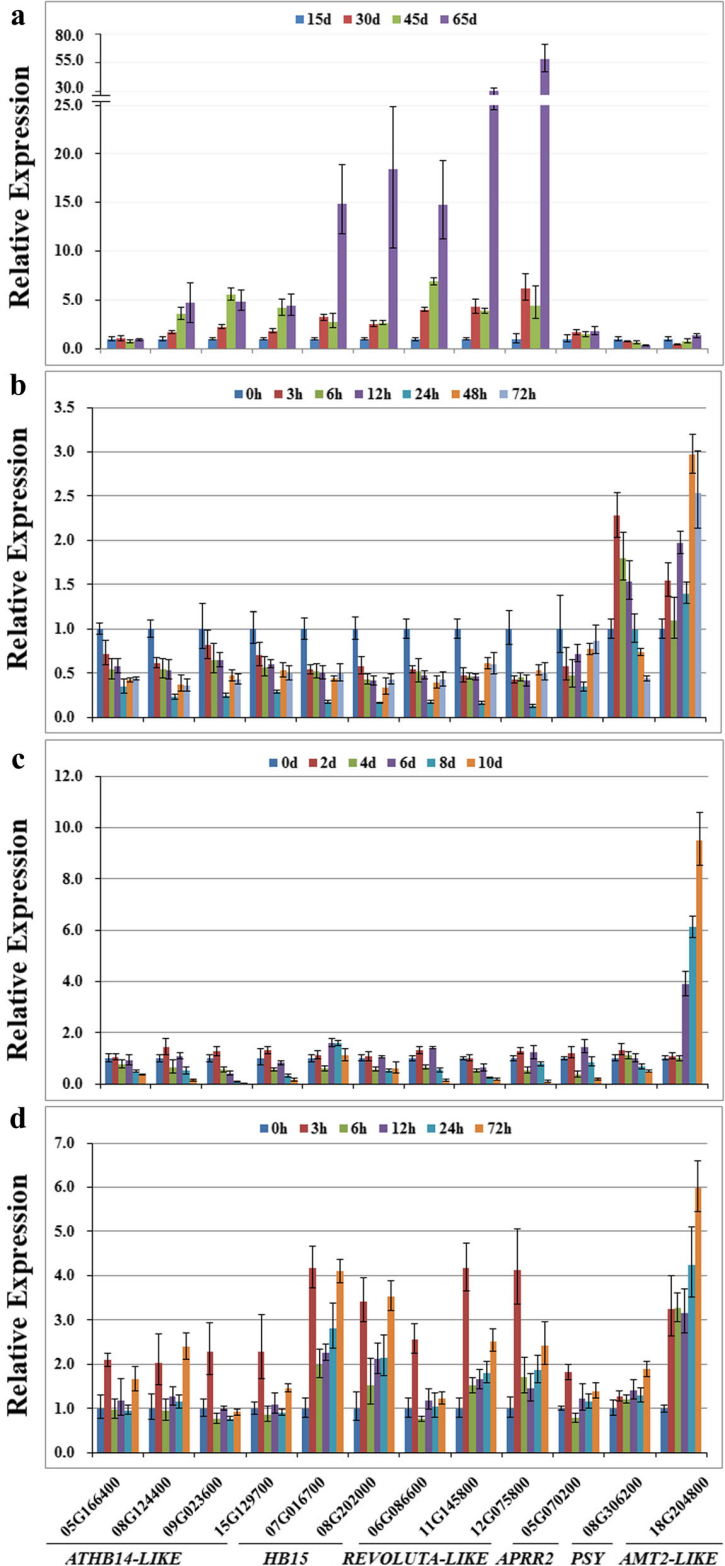
Expression analysis of the 12 target genes in response to seed development and abiotic stresses. Expression patterns of target genes during seed development (**a**), under cold stress (**b**), drought stress (**c**) and salinity stress (**d**). Stress treatments and seed development stage were used as same as the ones in Fig. 9. *PSY*, *PHYTOENE SYNTHASE*; *AMT2-LIKE*, *AMMONIUM TRANSPORTER 2-LIKE*. Error bars indicate SE of two biological and three technical replicates. Values were normalized against the *SUBI3* gene

To explore the regulatory function of miR166s in soybean, the expression patterns of 12 target genes with annotation in Phytozome were also analyzed by qRT-PCR during seed development and in response to abiotic stresses. As shown in Fig. 10a, the expressions of all the *HD-ZIP III* genes except Glyma05G166400 were gradually increased as seeds developed, and reached the peak at 65 DAF. Their fold changes ranged from 4.39 to 59.16, compared with the ones at 15 DAF. On the contrary, *PSY* was gradually decreased in transcript accumulation as seeds developed, while *AMT2-LIKE* was dramatically decreased by 2.34 folds at 30 DAF.

As indicated in Fig. 10b, the expressions of all the *HD-ZIP III* genes and *APRR2* were obviously decreased by cold stress, and reached their minimal levels at 24 h stress with 2.87–7.88 fold changes as compared to control. In contrast, the transcript accumulations of *PSY* and *AMT2-LIKE* were increased by 1.97 and 2.27 folds at 3 and 48 h after treatment, respectively.

Four *ATHB14-LIKE*, *REV-LIKE* (Glyma11G145800) and *PSY* displayed gradual decrease in transcript accumulation as drought stress prolonged (Fig. 10c), and their maximal fold changes ranged from 2.02 to 38.17. Although *ATM2-LIKE* displayed similar expression level to the control at early stage (2–4 days after drought treatment), the transcript accumulation was dramatically increased from 6 days after treatment until 10 days with 9.49 fold changes, suggesting that *ATM2-LIKE* might respond to drought stress at middle and late stages. The decreased transcript accumulation was also observed for the rest of target genes on one or more treatment points as compared to control.

As shown in Fig. 10d, all the *HD-ZIP III* genes and *APRR2* were promptly increased to their peaks in transcript accumulation at 3 h after salt stress with 1.82–4.17 fold changes, respectively as compared to control, indicating that they might be responsive to salt stress at early stage. The expressions of *PSY* and *AMT2-LIKE* were gradually increased, and reached their peaks at 72 h after salt treatment with 1.89–6.0 fold changes, respectively as compared to control.

## Discussion

miR166 acts as a highly conserved miRNA to be implicated in a wide range of cellular and physiological processes in plants [12, 14–18, 38]. Based on the data from miRBase, most of plant species harbor multiple miR166s. It has been accepted that the existence of multiple copies of *MIR* genes within plant species provides one possibility for functional redundancy and specificity [39]. Thus, the diversity and evolutionary conservation of *MIR166* family genes are expected to be elucidated for further understanding their fine-tuned regulatory roles in plant developmental processes and/or resistance to stresses.

Generally, miRNAs are annotated using comparative criteria for both their expression and biogenesis, including size and sequence of mature miRNA, phylogenetic conservation, secondary structure of miRNA precursor and increased precursor accumulation with decreased Dicer function in vivo [40]. In this study, the five newly identified miRNAs exhibited the same size and sequence as their paralogs in soybean (Table 1 and Fig. 2b). The lengths of the potential miRNA precursors range from 96 to 244 nt (Table 2 and Fig. 1), which is acceptable since the length of plant miRNA precursors has been found to be in the range of 55–930 nt (mean = ~146 nt) [30]. Furthermore, their precursors also satisfied the criteria with aspect to both phylogenetic conservation and secondary structure. It has been proposed that low MFE and high MFEI are reliable criteria to distinguish miRNAs from all coding and other non-coding RNAs, as these parameters, to certain extent, reflect the stability of the prefect or near-perfect secondary hairpin structure of miRNA precursor [31]. In the study, MFE values of the five newly identified pre-miR166s ranged from -46.0 to -90.7 kcal/mol, which were much lower than folding free energies of tRNA (-27.5 kcal/mol) and rRNA (-33 kcal/mol). At the same time, their individual MFEI value was also higher than that of tRNA, rRNA and mRNA. Taken together, the five newly identified miRNAs in soybean are very likely to be true miRNAs. Thus, the study enlarged the size of soybean miR166 family from 21 to 26 members.

It has been proposed that conserved *MIRNA* families have been both conserved and diversified during *MIRNA* evolution [39]. In this study, our data indicate that soybean *MIR166*s showed evolutionary conservation. As shown in Fig. 2a, ten pre-miR166 paralogous pairs with high sequence identity (93.8% or higher) were individually grouped into a discrete clade in the phylogenetic tree. Further analysis revealed that eight out of ten paralogous pairs were located at corresponding loci on the same duplication block (Fig. 3 and Additional file 7: Table S4), indicating that they might have arisen from segmental duplications without major sequence change. Additionally, smaller than 1 of Ka/Ks values suggest that these *MIR166* genes likely had undergone through a purifying selection with limited functional divergence after duplication. It is also worth noting that five paralogous pairs were clustered into discrete clades in phylogenetic tree based on their promoter sequences (Fig. 4), supporting the view that gene duplication could happen simultaneously at the coding region and the promoter [41]. Evidently, the origins of these soybean *MIR166*s were in line with soybean evolutionary history that soybean had undergone whole-genome duplication event ~56.5 million years ago [42]. Apart from the evolutionary conservation, soybean *MIR166*s also exhibited evolutionary diversification. In the study, five pre-miR166s (pre-miR166r, pre-miR166k and pre-miR166z; pre-miR166h and pre-miR166j) were separated far from the majority of pre-miR166s (Fig. 2a), and the identity between the five pre-miR166s and the rest of the pre-miR166s was low (40–66%) (Additional file 2: Figure S1). These observations suggested that they had diverged from the majority of *MIR166*s during soybean evolution.

Mature miR166s are ultimately responsible for functional diversification since they act as functional unit to regulate the expression of their targets. In the study, all the miR166s except miR166l were highly conserved in mature sequence, and identical mature sequence exists in each of the pre-miR166 paralogous pairs with exception of pre-miR166h and pre-miR166j (Fig. 2). Consequently, most of soybean miR166s shared the same putative targets (*HD-ZIP III* family) in soybean. However, miR166z, k, h, u, l are likely to have functional diversity since they are predicted to target genes other than those of the *HD-ZIP III* family, i.e., *APRR2*, *PHYTOENE SYNTHASE* and *AMMONIUM TRANSPORTER 2-LIKE* (Fig. 6a). Thus, our findings demonstrate that mature miR166s exhibit both functional conservation and diversification.

It has been well-known that miR166 plays important roles during seed development. For example, miR166 can repress the seed maturation program during vegetative development via controlling the expression of *HD-ZIP III* family genes such as *PHB* [14]; while it might also be involved in somatic embryo formation via acting as a positional signal from the basal–peripheral region of early embryos [13]. In this study, all the five newly identified *MIR166*s showed gradual increase during seed development, and reached their expression peaks at 65 DAF, a late stage of soybean seed development (Fig. 9a). These expression patterns are consistent with previous report that miR166 in *Brassica napus* was strongly accumulated at late stage of seed development [43]. Also, it was proposed that the accumulation of mature miR166 reached its major peak at late cotyledonary embryo stage in larch, suggesting that it might be associated with cotyledon formation [36]. The expression patterns of the five newly identified *MIR166*s suggested that they might be one of the major contributors to the network controlling seed development, especially seed maturation program.

It has been documented that plant miR166s might be involved in response to diverse abiotic stresses, and main evidences were derived from the altered accumulation pattern of mature miR166 under diverse abiotic stresses. For instance, the accumulation of mature miR166 was decreased by drought stress in *Sorghum bicolor* [38], whereas drought stress led to increase of miRN166 accumulation in root and leaf of wheat [37]. Another kind of examples are that miR166 in potato was significantly up-regulated by salinity stress [18], and depression of miR166 by small tandem target mimic can lead to improvement of salt tolerance in *Arabidopsis* [44]. In the study, the presence of stress-responsive elements in the promoter regions of *MIR166* genes pointed to their possible roles under various stresses (Fig. 4). Furthermore, the altered expression patterns of the five newly identified *MIR166* genes suggest their prominent roles in response to cold, drought and salinity (Fig. 9b-d). For example, *MIR166v* and *MIR166w* harbored the LTR cis-acting element in promoter regions, which can be involved in low-temperature responsiveness (Fig. 4). Indeed, *MIR166v* was promptly increased by cold stress with 7.79 fold changes, while *MIR166w* showed gradual increase in transcript accumulation as cold stress prolonged (Fig. 9b), implying that *MIR166v* and *MIR166w* can possibly function as a modulator of cold-induced signaling pathways. These results are consistent with previous reports that *MIR166e* and *MIR166r* were increased by chilling stress in vegetable soybean [45], and that the expression levels of *MIR166u* were obviously increased under cold condition in nitrogen-fixing nodules of soybean [46]. It has been proposed that spatial-temporal or specific expression of *MIRNA* isoforms is a crucial determinant of isoform specificity [13, 47]. In the study, the differential expression patterns of 5 *MIR166* genes under same stress indicated their functional specificity (Fig. 9b-d). As shown in Fig. 7c, *MIR166v* was dramatically decreased at 2 days after drought treatment, whereas *MIR166w* was increased at 4, 6, 8 days after drought treatment. Thus, the stress-specific properties of *MIR166* genes provided important clues for elucidating their functional specificity. Although five novel *MIR166*s in soybean were differentially regulated during seed development or in response to stresses, their expression patterns were not negatively correlated with the ones of miR166 target genes (Figs. 9 and 10). For example, the expressions of the *HD-ZIP III* genes were gradually increased as seeds developed. This is consistent with the previous report that *HD-ZIP III* family in *Arabidopsis* can be positively involved in regulation of seed mature program [14]. However, the five new identified *MIR166*s also showed gradual increase in transcript accumulations. These results suggested that miR166-mediated silencing of target genes was under sophisticated regulation. *MIR166* gene family has large number of members in plant species, and at least 26 miR166 members exist in soybean. So, we speculated that *MIR166* gene family possibly contributes to the accumulation pattern of target genes in a family member-specific manner.

It has been widely accepted that members of a certain evolutionary branch have potential to share similar functions [48]. In the study, pre-miR166x and pre-miR166y were the most closely related paralogs in the phylogenetic tree (Fig. 2a), and they also showed 99.0% sequence identity (Additional file 2: Figure S1). As expected, similar expression patterns were observed for *MIR166x* and *MIR166y* in response to cold, drought and salinity stresses (Fig. 9b-d). Additionally, the most closely related pre-miR166 paralogs (such as miR166h and miR166j; miR166g and miR166i) also shared similar tissue-specific expression pattern in soybean (Fig. 5). Thus, it was hypothesized that these most closely related pre-miR166 paralogs in soybean perform similar cellular functions during developmental processes or in response to various stresses.

## Conclusions

This study enlarged the size of soybean miR166 family from 21 to 26 members. A comprehensive analysis indicated that the 26 soybean miR166s exhibited evolutionary conservation and diversification. Furthermore, the expression patterns of five newly identified *MIR166*s during seed development and in response to abiotic stresses provided important clues for elucidating the functional specificity and redundancy of miR166 family in soybean. Future study will aim at investigating the effect of each *MIR166* gene on seed development and stress tolerance, identifying target genes and dissecting the regulatory mechanisms for each *MIR166*, and exploring combination of multiple *MIR166* genes during seed development and in response to specific stress.

## Methods

### Plant materials and treatments

Soybean (*Glycine max* cv. Williams 82) plants were grown at an experimental station in Jilin University (Changchun, Jilin Province, China), in 2015. Seeds were collected at 15, 30, 45, 65 days after flowering (DAF), and frozen in liquid nitrogen and stored at -80 °C. Mature seeds were also collected for the following experiments.

Six seeds were sown on each pot filled with 65 g vermiculite. All the seedlings were grown at 25 °C with a photoperiod (14 h light and 10 h dark) in a chamber, and regularly watered with Hoagland liquid medium. Ten-day-old seedlings were subjected to the following treatments, and six pots of plants were used for each treatment or control: (1) For cold stress, seedlings were transferred to 4 °C and samples were collected at 0, 3, 6, 12, 24, 48 and 72 h after cold treatment; (2) For drought stress, water supply was withheld and samples were collected at 0, 2, 4, 6, 8 and 10 days of water stress; (3) For salinity stress, 200 mM NaCl solution was applied to seedlings and samples were collected at 0, 3, 6, 12, 24 and 72 h after salt treatment. The above-ground parts were collected and frozen in liquid nitrogen, and stored at -80 °C.

### Identification of novel miR166s in soybean

Known miR165/166 precursor sequences from soybean, *Arabidopsis* and rice were downloaded from the publicly available miRBase (www.mirbase.org), and then used as reference sequences to search for their homologs against soybean genome in Phytozome (www.phytozome.net). Subsequently, these homologs were used in a BLAST search against NCBI nucleotide collection database and protein database to eliminate protein-encoding sequences and non-coding RNAs such as tRNA, rRNA, snRNA or snoRNA.

The candidates were then assessed for secondary structures, using RNA fold program (http://nhjy.hzau.edu.cn/kech/swxxx/jakj/dianzi/Bioinf4/miRNA/miRNA1.htm), and the parameters were set as default. Finally, the putative miRNAs were identified based on the following criteria [49]: 1) novel mature miR166 should be identical or nearly identical to one of known miR166s in plants; 2) the position of mature miRNA on the hairpin; 3) the maximum number of unpaired residues should be five between miRNA and miRNA*; 4) the maximum number of G_U pairs in miRNA should be 5; 5) the maximum size for a bulge in miRNA sequence should be 3 nt; 6) the negative minimal folding free energy (MFE) should be low; and 7) the minimal folding free energy index (MFEI) should be high. MFE denotes the negative folding free energies, and the minimal folding free energy Index (MFEI) was calculated by employing the following equation: MFEI = [(MFE/length of the RNA sequence) *100]/(G + C) %.

### Phylogenetic analysis of 26 miR166s and their precursors in soybean

Sequences of soybean miR166s and their precursors were collected from miRBase, and then aligned by ClustalW along with the ones of newly identified miR166s, respectively. Subsequently, phylogenetic trees were constructed by maximum likelihood method using software MEGA 5.2 [50], and the bootstrap value was calculated with 1000 replicates. Percentage identity of aligned sequences was calculated using Kalmogorov-Smirnov statistical test in GeneDoc.

### Chromosomal localizations and gene duplications

To determine the locations of the 26 pre-miR166s on soybean chromosomes, the coordinate of pre-miR166s and chromosome lengths were obtained from NCBI database. For syntenic mapping, we firstly used the coordinate of each pre-miR166 to map its genomic locus or flanking genes, which were subsequently subjected to retrieval of duplicated genomic regions and Ka/Ks values for each duplicated *MIR166*s from batch download option of Plant Genome Duplication Database (http://chibba.agtec.uga.edu/duplication/). Tandem duplications were defined as two paralogs separated by less than 3 kb in the same chromosome, while segmental duplications referred to those homologous genes distributed on duplicated chromosomal blocks from the same genome lineage. The chromosomal location image of soybean *MIR166* genes was generated by the Circos software [51].

### Promoter analysis of *MIR166* genes in soybean

The transcription start sites of *MIR166* genes were predicted by TSSP software in Softberry (http://linux1.softberry.com/berry.phtml?topic=tssp&group=programs&subgroup=promoter). For promoter analysis, a 1,500 bp interval upstream of the transcription start site of *MIR166* genes was analyzed in the PlantCARE database (http://bioinformatics.psb.ugent.be/webtools/plantcare/html/).

### Prediction of miR166 targets in soybean

The targets of soybean miR166s were predicted by using the psRNATarget program (http://plantgrn.noble.org/psRNATarget/) against the transcripts of *Glycine max* from JGI genomic project. The psRNATarget parameter settings were set as default with a maximum exception value of 3.

Unique miR166s (UmiR166s) and unique target sites of miR166s (UTSs) were identified according to the procedure described by Barik [52]. Briefly, the sequences of 262 mature miR166s from 45 plant species were aligned to isolate the UmiR166s according to their sequence similarity and uniqueness (Additional file 2: Table S2). Subsequently, the UmiR166 sequences were applied for the prediction of target genes from plant species using psRNATarget tool. The unique miR166 binding sites in target transcripts were designated as UTS and numbered (Additional file 10: Table S7). Phylogenetic trees were constructed by maximum likelihood method using software MEGA 5.2 [50].

RNA Ligase-Mediated RACE (RLM-RACE) was performed with the FirstChoice® RLM-RACE Kit (Ambion) according to the manufacturer’s instructions, with slight modification. Briefly, 10 μg of total RNA was directly ligated to the 5’ adaptor followed by reverse transcription with an oligo (dT) primer. PCR was performed with 5’ adaptor primers and 3’ gene-specific primers (Additional file 11: Table S8) using cDNA as the template. The RACE products were gel-purified, cloned, and sequenced.

### Tissue-specific expression of *MIR166*s and their target genes in soybean

Based on the coordinates of pre-miR166s on soybean genome in Phytozome, 11 pre-miR166s were mapped to the characterized genomic loci, and the expressions of the corresponding genes at these genomic loci were used to estimate the transcript level of *MIR166*s in different tissues. The fragments per kilobase of transcript per million mapped reads (FPKM) values for each *MIR166* and its targets were extracted from Phytozome database by tracking soybean gene-level expression (http://www.phytozome.net). The heatmap for *MIR 166* genes was generated in R using the heatmap.2 function from the gplots CRAN library (http://CRAN.R-project.org/package=gplots).

### Expression analysis of the newly identified pre-miR166s and target genes in soybean

Total RNA was isolated from seeds at different developmental stages and seedlings under different stress treatments using RNAprep Pure Plant Kit (Tiangen Inc, China). To examine the expression of pre-miRNAs, DNase-treated RNA of each sample was reverse transcribed to first-strand cDNA using pRimeScript RT reagent kit with gDNA Eraser (Takara) according to manufacturer’s instruction. qRT-PCR was subsequently conducted with an ABI 7500 PCR system and SYBR Premix Ex Taq (Takara), using *SUBI3* as internal control. Three replicates were performed for each sample, and data were analyzed by the software ABI 7500 v.20. Primer sequences are listed in Additional file 11: Table S8.

## Additional files

Additional file 1: Table S1. Characteristics of miR166 family in soybean. (XLSX 12 kb)
Additional file 2: Figure S1. Identity between pre-miR166s in soybean. (TIF 5906 kb)
Additional file 3: Table S2. Summary of 63 unique miR166s (UmiR166s) in plant species. (XLSX 12 kb)
Additional file 4: Figure S2. Phylogenetic analysis of all the unique miR166s (UmiR166s) in plant species. The tree is divided into four groups, and the seven UmiR166s presentative of 26 soybean miR166s are highlighted with a star. (TIF 649 kb)
Additional file 5: Figure S3. The percentage identity of the aligned miR166 sequences calculated using Kalmogorov-Smirnov statistical test in GeneDoc. Percentage identity of all mature miR166 sequences **(A)** and their precursor sequences **(B)** in plant species. (TIF 355 kb)
Additional file 6: Table S3. Corresponding gene or EST of pre-miR166s in soybean. (DOCX 21 kb)
Additional file 7: Table S4. Segmental duplication events during evolution of MIR166 family genes in soybean. (XLSX 13 kb)
Additional file 8: Table S5. Transcription start site of *MIR166* genes in soybean. (DOCX 19 kb)
Additional file 9: Table S6. Promoter analysis of MIR166 family genes in soybean. (XLSX 19 kb)
Additional file 10: Table S7. Summary of UTSs and targets of miR166s. (XLSX 19 kb)
Additional file 11: Table S8. Primers used for Real-time PCR. (XLSX 13 kb)

## Abbreviations

miR166: microRNA166
pre-miRNA: Stem-loop precursor of miRNA
miRISC: miRNA-induced silencing complex
*RDD1*: *RICE Dof DAILY FLUCTUATIONS 1*
MFE: Minimal folding free energy
MFEI: Minimal folding free energy index
EST: Expressed Sequence Tags
Ka/Ks: Non-synonymous/synonymous substitution ratio
FPKM: Fragments per kilobase of transcript per million mapped reads
DAF: Days after flowering
RLM-RACE: RNA Ligase-Mediated RACE
