# Supplementary figures and images for: Conservation and diversification of the miR166 family in soybean and potential roles of newly identified miR166s
Source: BMC Plant Biol. 2017 Feb 1;17:32. doi: 10.1186/s12870-017-0983-9 (PMC5286673; doi:10.1186/s12870-017-0983-9)

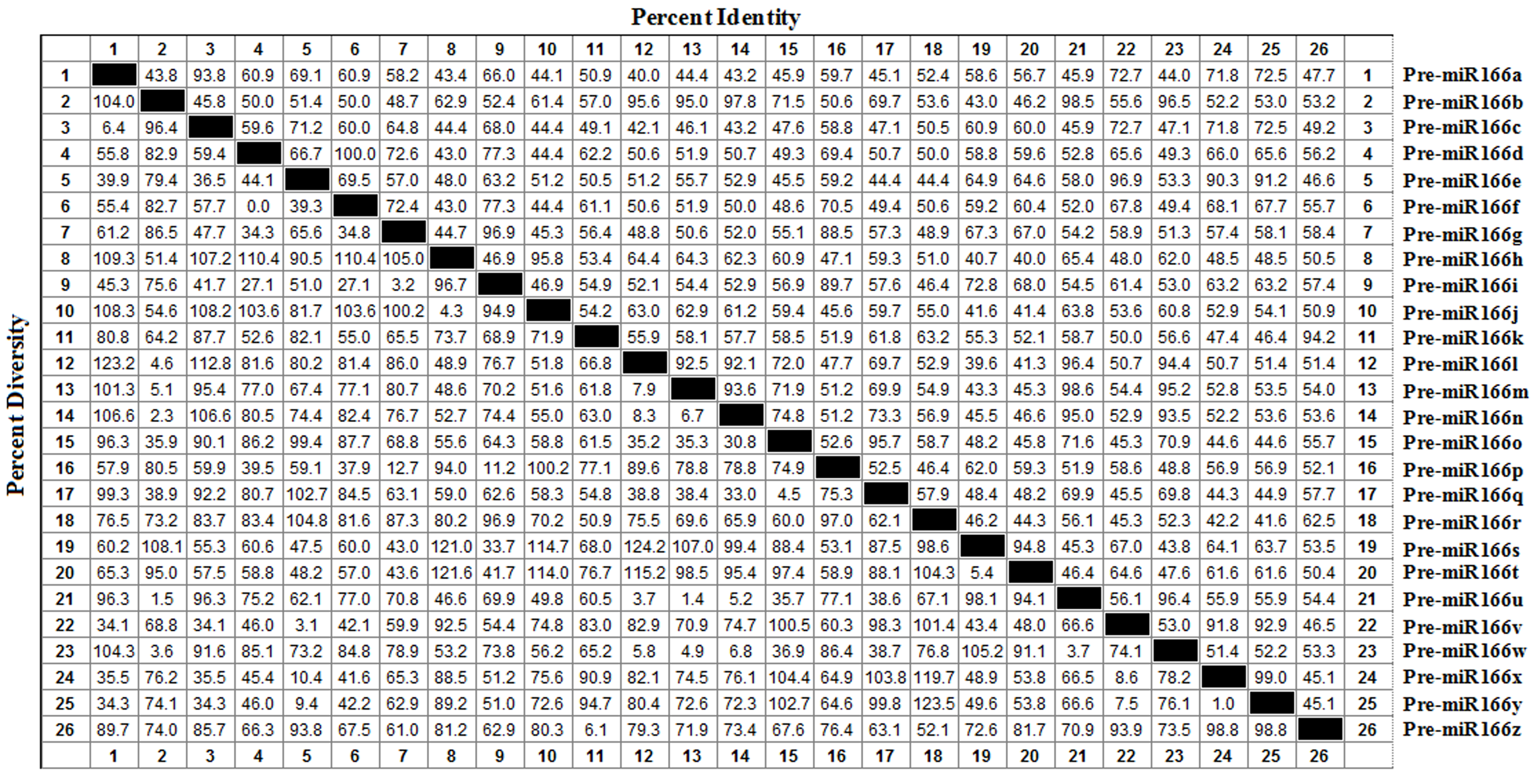

Supplement: Additional file 2: Figure S1. — Identity between pre-miR166s in soybean. (TIF 5906 kb) [file 12870_2017_983_MOESM2_ESM.tif]

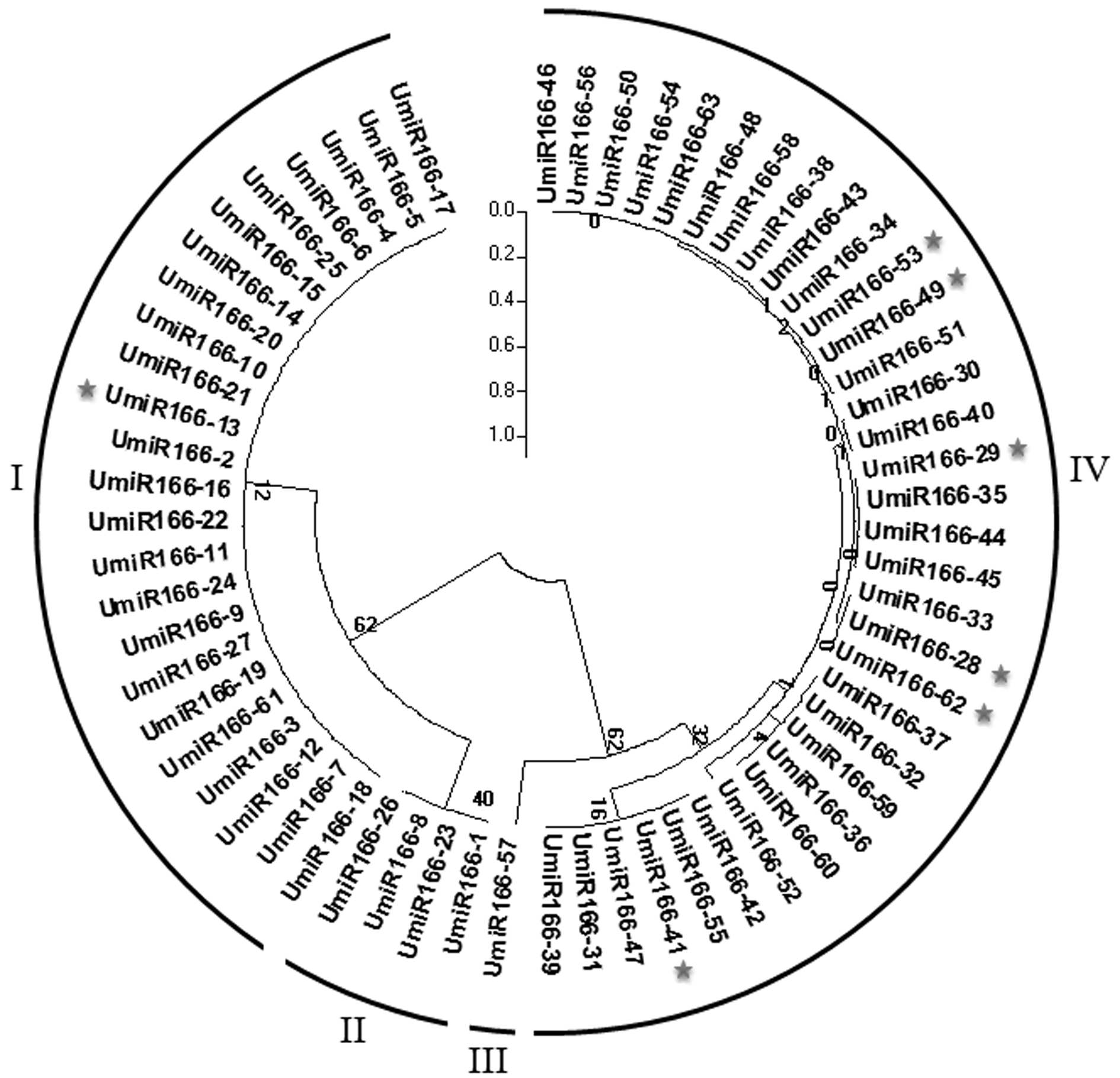

Supplement: Additional file 4: Figure S2. — Phylogenetic analysis of all the unique miR166s (UmiR166s) in plant species. The tree is divided into four groups, and the seven UmiR166s presentative of 26 soybean miR166s are highlighted with a star. (TIF 649 kb) [file 12870_2017_983_MOESM4_ESM.tif]

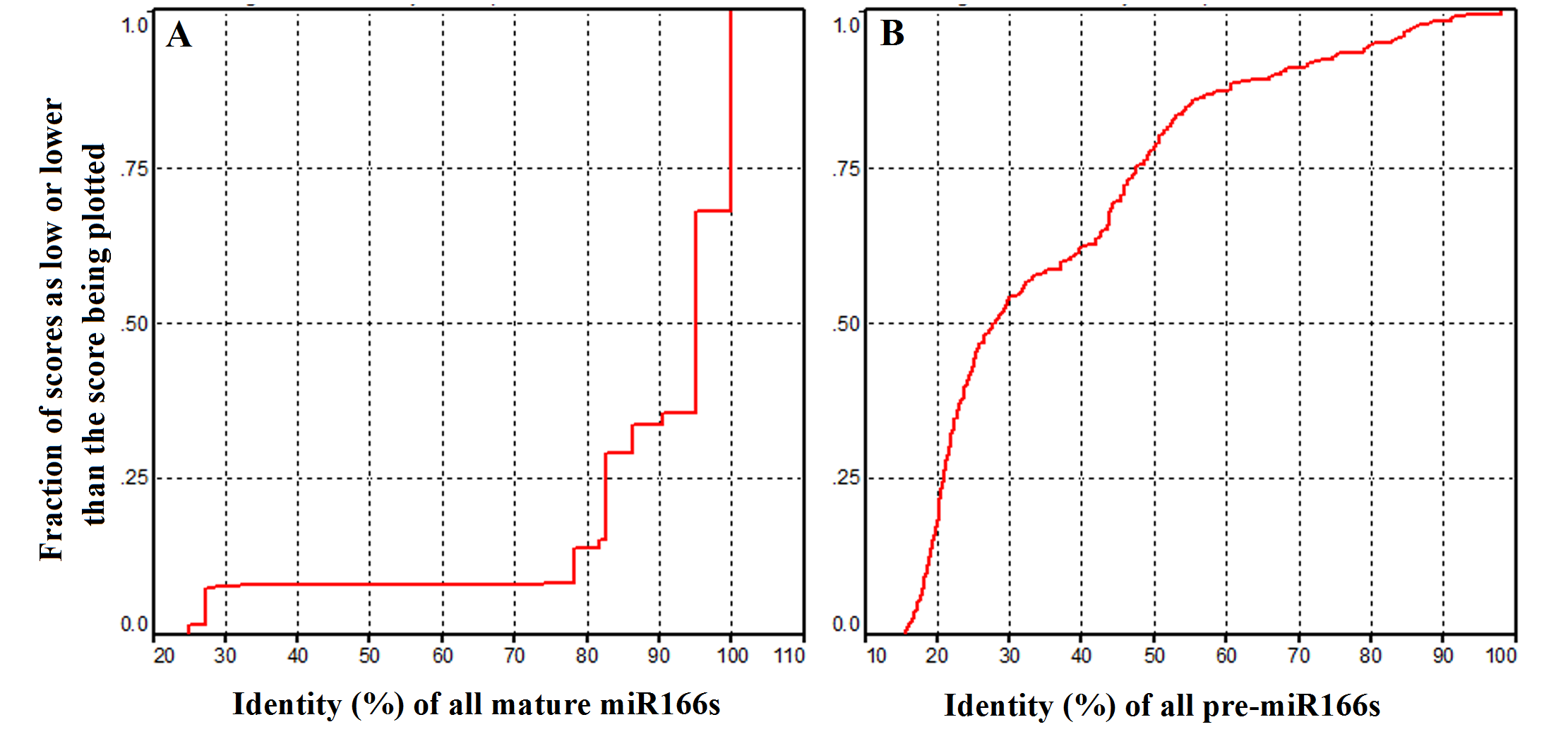

Supplement: Additional file 5: Figure S3. — The percentage identity of the aligned miR166 sequences calculated using Kalmogorov-Smirnov statistical test in GeneDoc. Percentage identity of all mature miR166 sequences (A) and their precursor sequences (B) in plant species. (TIF 355 kb) [file 12870_2017_983_MOESM5_ESM.tif]
