# Supplementary material for: Conservation and diversification of the miR166 family in soybean and potential roles of newly identified miR166s
Source: BMC Plant Biol. 2017 Feb 1;17:32. doi: 10.1186/s12870-017-0983-9 (PMC5286673; doi:10.1186/s12870-017-0983-9)
Supplement: Additional file 6: Table S3. — Corresponding gene or EST of pre-miR166s in soybean. (DOCX 21 kb) [file 12870_2017_983_MOESM6_ESM.docx]

**Table S3** Corresponding gene or EST of pre-miR166s in soybean.

| **ID** | **Type** | **Locus name** | **Location on gene** | **Corresponding EST** | **Resource of ESTs** |
| --- | --- | --- | --- | --- | --- |
| Pre-miR166a | Intragenic | Glyma.16G020900 | 5'UTR and 1st exon (+) | BI893541  (19-164) | Drought stressed leaf |
| Pre-miR166e | Intragenic | Glyma.04G207000 | 5'UTR and 1st exon (+) | HO040431  (52-161) | Immature seed |
| Pre-miR166g | Intragenic | Glyma.10G033700 | 1st exon (+) | N/A | N/A |
| Pre-miR166h | Intragenic | Glyma.08G186300 | 5'UTR, 1st exon,1st intron, 2st exon (+) | N/A | N/A |
| Pre-miR166i | Intragenic | Glyma.02G140300 | 1st exon (+) | BQ785760  (1-72) | Wounded cotyledons |
| Pre-miR166j | Intragenic | Glyma.15G046500 | 5'UTR (+) | BI972515  (165-332) | Germinating shoots |
| Pre-miR166n | Intragenic | Glyma.05G196200 | 5'UTR and 1st exon (+) | EV280596  (272-414) /HO034798  (280-442) | Root and stressed root / Immature seed |
| Pre-miR166p | Intragenic | Glyma.03G159900(*UNAG KINASE*) | 5'UTR (+) | N/A | N/A |
| Pre-miR166q | Intragenic | Glyma.04G207000 | 3'UTR (+) | HO040431  (309-460) | Immature seed |
| Pre-miR166y | Intragenic | Glyma.05G196200 | 5'UTR (+) | EV280596  (18-119) | Root and stressed root |
| Pre-miR166z | Intragenic | Glyma.16G039100 | 5'UTR (+) | N/A | N/A |
| Pre-miR166r | Intragenic | Glyma06G13900 | 5'UTR,1st exon (-) | FK007014  (136-290) | Stressed tissues |
| Pre-miR166b | Intragenic | Glyma.08G004000 | 1st exon (-) | HO020191  (222-338) | Immature seed |
| Pre-miR166c | Intragenic | Glyma.07G052000 | 1st exon (-) | N/A | N/A |
| Pre-miR166l | Intragenic | Glyma.06G268300(*CHAPERONIN*) | 3st intron (-) | N/A | N/A |
| Pre-miR166x | Intergenic | N/A | N/A | HO020191  (30-132) | Immature seed |
| Pre-miR166v | Intergenic | N/A | N/A | HO028458  (26-124) | Immature seed |
| Pre-miR166o | Intergenic | N/A | N/A | HO028458  (261-405) | Immature seed |
| Pre-miR166d | Intergenic | N/A | N/A | N/A | N/A |
| Pre-miR166f | Intergenic | N/A | N/A | N/A | N/A |
| Pre-miR166k | Intergenic | N/A | N/A | N/A | N/A |
| Pre-miR166m | Intergenic | N/A | N/A | N/A | N/A |
| Pre-miR166s | Intergenic | N/A | N/A | N/A | N/A |
| Pre-miR166t | Intergenic | N/A | N/A | N/A | N/A |
| Pre-miR166u | Intergenic | N/A | N/A | N/A | N/A |
| Pre-miR166w | Intergenic | N/A | N/A | N/A | N/A |

(+) and (-) refer to sense strand and anti-sense strand of miR166 corresponding gene, respectively.
