# Supplementary material for: Conservation and diversification of the miR166 family in soybean and potential roles of newly identified miR166s
Source: BMC Plant Biol. 2017 Feb 1;17:32. doi: 10.1186/s12870-017-0983-9 (PMC5286673; doi:10.1186/s12870-017-0983-9)
Supplement: Additional file 8: Table S5. — Transcription start site of MIR166 genes in soybean. (DOCX 19 kb) [file 12870_2017_983_MOESM8_ESM.docx]

| ID | **Chromosome** | **Strand** | **Predicted TSS** | **Distance between pre-miRNA and TTS** | **Distance between TATA Box and TTS** |
| --- | --- | --- | --- | --- | --- |
| *MIR166a* | Chr16 | - | 1913590 | 642 | 100 |
| *MIR166b/x* | Chr08 | - | 283175 | 399 | 35 |
| *MIR166c* | Chr07 | - | 4453995 | 213 | 78 |
| *MIR166d* | Chr20 | - | 43105555 | 55 | 59 |
| *MIR166e/q* | Chr04 | + | 44848776 | 787 | 9 |
| *MIR166f* | Chr10 | + | 41243307 | 51 | 58 |
| *MIR166g* | Chr10 | - | 2907325 | 1711 | 12 |
| *MIR166h* | Chr08 | + | 14989085 | 1254 | 9 |
| *MIR166i* | Chr02 | + | 14336587 | 3973 | 94 |
| *MIR166j* | Chr15 | - | 3689306 | 177 | 7 |
| *MIR166k* | Chr19 | - | 36650159 | 298 | 27 |
| *MIR166l* | Chr06 | - | 45112317 | 1217 | 21 |
| *MIR166m* | Chr09 | + | 33908706 | 167 | 33 |
| *MIR166n/y* | Chr05 | - | 37748454 | 871 | 10 |
| *MIR166o/v* | Chr06 | - | 12993929 | 712 | 12 |
| *MIR166p* | Chr03 | - | 39520210 | 188 | 27 |
| *MIR166r* | Chr06 | + | 10985362 | 234 | 29 |
| *MIR166s* | Chr07 | + | 10198726 | 95 | 50 |
| *MIR166t* | Chr09 | - | 37128604 | 3224 | 338 |
| *MIR166u* | Chr04 | + | 25371659 | 1215 | 33 |
| *MIR166w* | Chr01 | - | 26216471 | 624 | 408 |
| *MIR166z* | Chr16 | + | 3661106 | 265 | 25 |

**Table S5** Transcription start site of *MIR166* genes in soybean
